# Psychological resilience buffers the association between cell phone addiction and sleep quality among college students in Jiangsu Province, China

**DOI:** 10.3389/fpsyt.2023.1105840

**Published:** 2023-02-08

**Authors:** Guangming Xie, Qi Wu, Xiaohan Guo, Jinpeng Zhang, Dehui Yin

**Affiliations:** ^1^School of Medical Imaging, Xuzhou Medical University, Xuzhou, China; ^2^Key Laboratory of Human Genetics and Environmental Medicine, School of Public Health, Xuzhou Medical University, Xuzhou, China

**Keywords:** cell phone addiction, psychological resilience, sleep quality, Pittsburgh Sleep Quality Index, mobile phone addiction

## Abstract

**Background and aims:**

Excessive use of cell phones can take up college students’ time and energy, and the sleep quality will inevitably be affected. A high level of psychological resilience can help them to maintain a positive attitude and cope with stressful events. However, few studies were conducted to investigate the effects of psychological resilience buffering cell phone addiction on sleep quality. In our hypothesis, psychological toughness would mitigate the worsening impact of cell phone addiction on sleep quality.

**Methods:**

The sample consisted of 7,234 Chinese college students who completed an electronic questionnaire that included demographic characteristics, such as the Mobile Phone Addiction Index (MPAI), the Psychological Resilience Index (CD-RISC), and the Pittsburgh Sleep Quality Index (PSQI). SPSS 26.0 was used for data analysis, the measurement data were described by $\bar{x}$ ± s for those conforming to normal distribution, and the comparison of means between groups was analyzed by group *t*-test or one-way ANOVA. Those that was not conforming to normal distribution were described by median *M* (*P*_25_, *P*_75_), and the comparison of *M* between groups was analyzed by Mann–Whitney *U* test and Kruskal–Wallis *H* test. Using Spearman correlation analysis, the associations between mobile phone addiction, psychological resilience, and sleep quality were evaluated. Using SPSS Process, the mediating role of psychological resilience was examined.

**Results:**

The mean scores of cell phone addiction and psychological resilience were 45.00 (*SD* = 13.59) and 60.58 (*SD* = 18.30), respectively; the sleep quality score *M* (*P*_25_, *P*_75_) was 5.0 (3.0, 7.0). Cell phone addiction among college students had a direct predictive effect on sleep quality (β = 0.260, *P* < 0.01), and psychological resilience had a negative correlation with both cell phone addiction and sleep quality (β = –0.073, *P* < 0.01, and β = –0.210, *P* < 0.01). Psychological resilience was responsible for a mediating effect value of 5.556% between cell phone addiction and sleep quality.

**Conclusion:**

Cell phone addiction has an impact on sleep quality both directly and indirectly through the mediating effect of psychological resilience. Increased psychological resilience has the potential effect to buffer the exacerbating of cell phone addiction on sleep quality. These findings provide an evidence for cell phone addiction prevention, psychological management, and sleep improvement in China.

## Introduction

Sleep is a fundamental physiological and psychological process for human health (1), and adequate sleep plays a crucial role in developing physical, cognitive, and psychological functions in different populations (2, 3). On the other hand, poor sleep may pose a serious threat to some psychological, physical, and social functioning aspects of individuals, such as cognitive ability, mood, immunity, quality of life, wellbeing, learning, and social interaction (4). It can lead to depression and anxiety (5) and behaviors such as smoking and alcohol consumption (6). Studies have shown that the prevalence of poor sleep quality and disorders among Chinese university students is 30% (7) and 25.7%, respectively (8). The China Sleep Research Report 2022, published by the Chinese Academy of Social Sciences on World Sleep Day, reported that more than 90% of Chinese college students had an average (49%) and poor (45%) sleep quality, and more than half of them felt that they did not have enough sleep time. Most of students had the problem of “sleep procrastination.” Sleep is affected by college students’ lifestyle habits, study pressure, and cell phone dependence (9). In addition, study found that approximately 96% of USA undergraduate students reported poor sleep quality (global PSQI score ≥ 6) (10).

College students face some behavioral problems, one of which is cell phone addiction (11). Some researchers have found that young people are more attracted to the latest technology than older people, and smartphones are more attractable to younger generation (12). According to published studies, excessive use of cell phones may lead to physical and mental health problems (13–15). Excessive cell phone use can also lead to addiction and may be associated with sleep disorders (16–18). Therefore, it would be interesting to investigate the relationship between cell phone use and sleep quality among college students. According to the emotion processing model of negative reinforcement, a major reason for maintaining cell phone addiction is the inability of people with problematic cell phone use to deal with the negative emotions that accompany addiction (19). Similarly, several studies have proposed that it is due to individuals’ shortages in regulating negative emotions that cell phone addiction continues to be maintained (20).

Mental health problems among college students are also concerned in the face of pressures related to education, romantic relationships, and employment. One behavior that might harm someone’s mental health and encourage dangerous, but can be buffered by psychological resilience. In theory, psychological resilience includes process, dynamic, competency, and outcome dimensions (21). Psychological resilience is a character quality that protects people from adverse or traumatic experiences, following the paradigm of positive psychology and its calming influence on troublesome behaviors (22). Additionally, psychological resilience places a high value on the potential strength of individuals and pertinent contextual elements, and also helps to shape an individual’s entire growth.

There is evidence that individuals with high psychological resilience have better psychological wellbeing, including higher self-regulation skills, higher self-esteem, and more significant social support. In addition, an individual’s psychological resilience may play an important role in preventing the emergence of problem behaviors (23). Several studies have found that psychological resilience is negatively associated with cell phone addiction (24, 25), as well as some behaviors such as alcohol abuse and eating disorders (26, 27). Based on these studies, psychological resilience may play an important role in preventing the emergence of excessive behaviors.

A prospective study reported that sleep quality was bilaterally associated with psychological resilience, and people with higher psychological resilience had fewer sleep disturbances and shorter sleep latency (28). In addition, a study of adults in the community showed that subjective stress was a risk factor for sleep disorders, while psychological resilience played a protective role against sleep disorders. Furthermore, there was an interaction effect between subjective stress and psychological resilience, with psychological resilience buffering the adverse impacts of subjective stress on sleep (29). Zhou et al. analyzed a study of a college student population and found that psychological resilience may buffer sleep quality on the exacerbating effect of depressive symptoms. According to their findings, the impact of psychological resilience on sleep quality cannot be ignored (30).

However, to date, no studies have explored the role of emotion regulation (e.g., psychological resilience) in the relationship between cell phone addiction and sleep quality. Therefore, this study aimed to explore the buffering capacity of psychological resilience between mobile phone addiction and sleep quality. We proposed two hypotheses:

Hypothesis 1: Psychological resilience, cell phone addiction, and sleep quality of college students are associated with each other.

Hypothesis 2: Psychological resilience of college students mediates the relationship between cell phone addiction and sleep quality.

**FIGURE 1:**
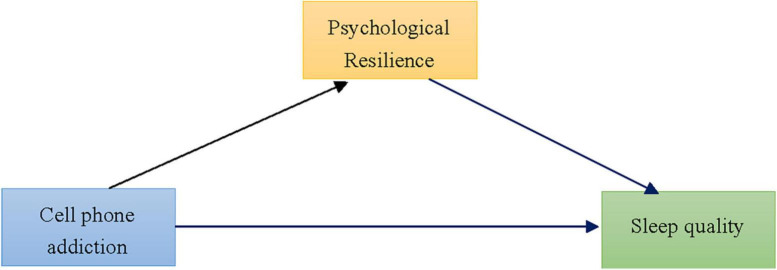
The proposed mediation model.

Based on previous studies, the purpose of this study wasto examine the effect of cell phone addiction on sleep quality, where psychological resilience was the mediating variable. It was hypothesized that cell phone addiction would be related to sleep quality; psychological resilience would mediate the association between cell phone addiction and sleep quality (Figure 1).

## Materials and methods

### Participants

An electronic questionnaire-based survey was conducted on the Questionnaire Wenjuanxing platform^1^ from October 2022 to November 2022. Participants included 7,363 college students (mean age = 18.86 years; *SD* ± 2.6) through a whole-group convenience sampling from universities in Jiangsu Province, China. Due to incomplete information, 129 participants were excluded from the data set. The final sample set consisted of 7,234 participants (2,157 males and 5,077 females) with a response rate of 98.25%.

### Measures

#### Pittsburgh Sleep Quality Index (PSQI)

Sleep quality was assessed using the Pittsburgh Sleep Quality Index (PSQI) (31). Sixteen of the 18 self-rated questions involved in scoring this scale were scored separately (removing the two questions on bedtime and wake-up time), consisting of seven dimensions of sleep onset, sleep duration, sleep efficiency, sleep disturbance, subjective sleep quality, hypnotic medication, and daytime dysfunction, rated over the last 1 month. The scores of the seven components are summed to produce a PSQI score, where the total score ranges from 0 to 21 (0–3 for each component), with higher scores indicating poorer sleep quality. The threshold for sleep disturbance is 7. In this study, the Cronbach’s alpha for the PSQI was 0.877, and the Kaiser-Meyer-Olkin value was 0.926.

#### Mobile phone addiction index (MPAI)

Smartphone addiction was assessed using the Mobile Phone Addiction Index (MPAI) (32), which was translated into Chinese and is widely used, validated for Chinese population (33, 34). The MPAI contains 17 items assessing four domains: inability to control craving, feeling anxious and lost, withdrawal/escape, and productivity loss. Each item is responded to from 1 (“not at all”) to 5 (“always”). Final scores were summed, and higher total scores reflect higher levels of smartphone addiction. In this study, the Cronbach’s alpha for the MPAI was 0.925, and the Kaiser-Meyer-Olkin value was 0.919.

#### Connor-Davidson Resilience Scale (CD-RISC)

The Connor-Davidson Resilience Scale (CD-RISC) (22) was chosen for this study to measure the level of psychological resilience in college students. The scale has now been developed into several versions and is widely used in different populations and in different situations (35–37). The CD-RISC contains 25 items with responses ranging from 5 points for all items as follows: not true at all (0), rarely true (1), sometimes true (2), usually true (3), and true almost all the time (4). This scale was rated based on how the participant has felt over the past month. The total score ranges from 0 to 100, with a higher score indicating more resilience. In this study, the Cronbach’s alpha for the CD-RISC was 0.970, and the Kaiser-Meyer-Olkin value was 0.979.

### Statistical analysis

SPSS 26.0 was used for statistical analysis. All data were tested for normal distribution using Shapiro–Wilk. The data were described by $\bar{x}$ ± s for those who met the normal distribution by normality test; *t*-test was used to compare the means between two groups; one-way ANOVA was used to compare the means between multiple groups. The data that did not meet the normal distribution by normality test were described by *M* (*P*_25_, *P*_75_), and the Mann–Whitney *U* test was used for the comparison of *M* between two groups, and the Kruskal–Wallis *H* test was used for the comparison of *M* between multiple groups. Correlations between factors were analyzed using Spearman correlation analysis. Next, we tested the mediating effect of psychological resilience between cell phone addiction and sleep quality using the Bootstrap method (Model 4) with repeated random sampling 5,000 times in the original data to calculate 95% confidence intervals (CI) for the mediating effect. A path is significant if the 95% confidence interval does not include 0. *P-*values under 0.05 are accepted as statistically significant results.

## Results

### Common method biases

To avoid common method biases, this study was carried out by collecting anonymous responses and scoring some items in reverse. The Harman’s single-factor test was used to conduct exploratory factor analysis on 60 items of the 3 scales, and a total of 10 factors had a characteristic root greater than 1. The variance explained by the first factor was 26.009%, which was less than the critical criterion of 40%. Therefore, the there was no serious common method bias in this study.

### The prevalence of sleep disturbance

Based on 7 as the threshold value for the PQSI, of the 7,234 participants, 6,043 (83.5%) reported normal sleep quality, and 1,191 (16.5%) reported sleep disturbance.

### Cell phone dependence, psychological resilience, and sleep quality scores of college students with different characteristics

The MPAI, CD-RISC, and PSQI scores of different groups among 7,234 college students are shown in Tables 1–3.

**TABLE 1:** Mobile phone addiction scores of college students with different characteristics.

Variable		*n* (%)	MPAI ($\bar{x}$ ± s)	F/t	*P*
Gender	Male	2157 (29.82)	42.45 ± 14.57	−10.476	< 0.001
	Female	5077 (70.18)	46.09 ± 13.01		
Grade	First year	4585 (63.38)	44.86 ± 13.38	3.424	0.033
	Second year	2270 (31.38)	45.50 ± 13.72		
	Third year or more	379 (5.24)	43.74 ± 15.15		
Native place	Urban	2847 (39.36)	44.97 ± 13.79	−0.163	0.871
	Rural	4387 (60.64)	45.02 ± 13.466		
Only child or not	Yes	2756 (38.10)	44.81 ± 14.19	−0.940	0.347
	No	4478 (61.90)	45.12 ± 13.21		
Smoking	Never	6871 (94.98)	44.96 ± 13.48	9.128	< 0.001
	Occasional	269 (3.72)	44.10 ± 13.55		
	Often	94 (1.30)	50.78 ± 19.35		
Drinking	Never	4973 (68.74)	44.55 ± 13.65	12.958	< 0.001
	Occasional	2176 (30.08)	45.82 ± 13.13		
	Often	85 (1.18)	50.21 ± 19.04		
Physical exercise	≤ 1 per month	1087 (15.03)	47.38 ± 14.74	68.864	< 0.001
	1–3 times per week	5020 (69.39)	45.39 ± 12.98		
	4–7 times per week	1127 (15.58)	41.00 ± 14.29		

**TABLE 2:** Psychological resilience scores of college students with different characteristics.

Variable		*n* (%)	CD-RISC ($\bar{x}$ ± s)	F/t	*P*
Gender	Male	2157 (29.82)	62.59 ± 21.06	6.117	< 0.001
	Female	5077 (70.18)	59.27 ± 16.92		
Grade	First year	4585 (63.38)	61.26 ± 18.26	9.302	< 0.001
	Second year	2270 (31.38)	59.54 ± 17.96		
	Third year or more	379 (5.24)	58.53 ± 20.30		
Native place	Urban	2847 (39.36)	62.43 ± 19.29	6.809	< 0.001
	Rural	4387 (60.64)	59.38 ± 17.52		
Only child or not	Yes	2756 (38.10)	62.07 ± 19.44	5.430	< 0.001
	No	4478 (61.90)	59.66 ± 17.50		
Smoking	Never	6871 (94.98)	60.72 ± 18.11	4.653	0.01
	Occasional	269 (3.72)	57.31 ± 19.21		
	Often	94 (1.30)	59.53 ± 26.85		
Drinking	Never	4973 (68.74)	60.77 ± 18.08	1.450	0.235
	Occasional	2176 (30.08)	60.24 ± 18.32		
	Often	85 (1.18)	58.06 ± 27.95		
Physical exercise	≤ 1 per month	1087 (15.03)	56.06 ± 19.23	57.054	< 0.001
	1–3 times per week	5020 (69.39)	60.73 ± 17.14		
	4–7 times per week	1127 (15.58)	64.26 ± 21.27		

**TABLE 3:** Sleep quality scores of college students with different characteristics.

Variable		*n* (%)	PSQI *M* (*P*_25_, *P*_75_)	*Z*/*H*	*P*
Gender	Male	2157 (29.82)	4.5 (3.0, 6.0)	4.293	< 0.001
	Female	5077 (70.18)	4.9 (3.0, 7.0)		
Grade	First year	4585 (63.38)	4.5 (3.0, 6.0)	80.017	< 0.001
	Second year	2270 (31.38)	5.1 (3.0, 7.0)		
	Third year or more	379 (5.24)	5.3 (4.0, 7.0)		
Native place	Urban	2847 (39.36)	4.6 (3.0, 6.0)	4.936	< 0.001
	Rural	4387 (60.64)	4.9 (3.0, 7.0)		
Only child or not	Yes	2756 (38.10)	4.5 (3.0, 6.0)	5.026	< 0.001
	No	4478 (61.90)	4.9 (3.0, 7.0)		
Smoking	Never	6871 (94.98)	4.7 (3.0, 6.0)	57.078	< 0.001
	Occasional	269 (3.72)	5.6 (4.0, 7.0)		
	Often	94 (1.30)	7.0 (4.0, 11.3)		
Drinking	Never	4973 (68.74)	4.6 (3.0, 6.0)	56.068	< 0.001
	Occasional	2176 (30.08)	5.0 (3.0, 7.0)		
	Often	85 (1.18)	6.6 (4.5, 9.0)		
Physical exercise	≤ 1 per month	1087 (15.03)	5.3 (3.0, 7.0)	51.414	< 0.001
	1–3 times per week	5020 (69.39)	4.7 (3.0, 6.0)		
	4–7 times per week	1127 (15.58)	4.5 (3.0, 6.0)		

### Analysis of the correlation between cell phone addiction, sleep disorder, and psychological resilience among college students

The correlation analysis showed that: cell phone addiction of college students was significantly and positively correlated with sleep quality (*r*_*s*_ = 0.248, *P* < 0.001); cell phone addiction of college students was significantly and negatively correlated with psychological resilience (*r*_*s*_ = –0.132, *P* < 0.001). There was a significant negative correlation between psychological resilience and sleep quality among college students (*r*_*s*_ = –0.231, *P* < 0.001) (Table 4).

**TABLE 4:** Correlation analysis of cell phone addiction, sleep disorder, and psychological resilience among college students (*n* = 7234).

	Cell phone addiction	Psychological resilience	Sleep quality
Cell phone addiction	1.000		
Psychological Resilience	–0.132***	1.000	
Sleep quality	0.248***	–0.231***	1.000

***P < 0.001.

### Testing of the mediation effects from cell phone addiction to sleep quality *via* psychological resilience

Using sleep quality as the dependent variable, cell phone addiction as the independent variable, and psychological flexibility as the mediating variable. Model 4 in the macro program developed by Hayes was used to test for mediating effects. Based on the bias-corrected percentile Bootstrap method, 95% confidence intervals for the various effects were estimated by sampling 5000 Bootstrap samples, and the mediating effects were significant if the confidence intervals did not include 0.

The results showed that the positive predictive effect of cell phone addiction on sleep quality was significant (β = 0.260, *t* = 22.854, *P* < 0.001) and the direct predictive effect of cell phone addiction on sleep quality decreased after putting in mediating variables, but it was still significant (β = 0.246, *t* = 21.999, *P* < 0.001). The negative predictive effect of cell phone addiction on psychological resilience was significant (β = –0.073, *t* = –6.217, *P* < 0.001). The negative predictive effect of psychological resilience on sleep quality was also significant (β = –0.192, *t* = 17.192, *P* < 0.001) (Table 5).

**TABLE 5:** Regression analysis of psychological flexibility on cell phone addiction and sleep quality among college students.

Step	Y	X	*R*	*R* ^2^	*F*	β	*t*
Step 1	Sleep quality	Cell phone addiction	0.260	0.067	522.307***	0.260	22.854***
Step 2	Psychological Resilience	Cell phone addiction	0.073	0.005	38.656***	-0.073	-6.217***
Step 3	Sleep quality	Cell phone addiction	0.322	0.104	419.578***	0.246	21.999***
		Psychological Resilience				-0.192	-17.192***

X is the independent variable, Y is the dependent variable.

**FIGURE 2:**
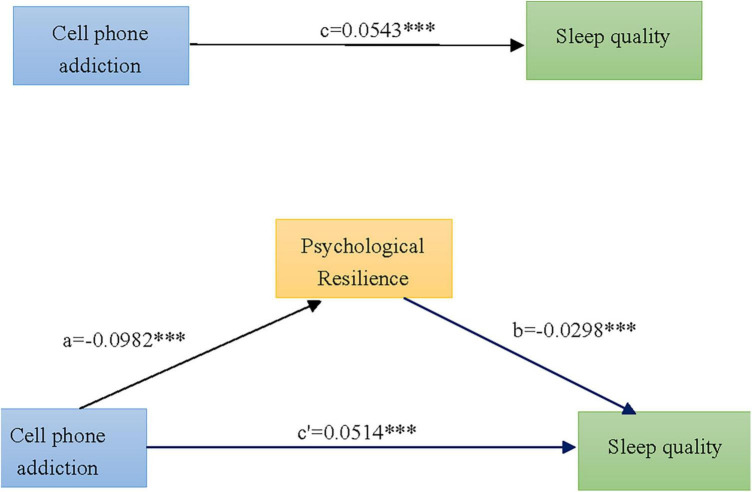
Mediating effect of psychological resilience between cell phone addiction and sleep quality among 7,234 college students. c-Total effect of cell phone addiction on sleep quality; a-Effect of cell phone addiction on the mediating variable psychological flexibility; b-Effect of psychological flexibility on sleep quality after controlling for the effect of cell phone addiction; c′-Direct effect of cell phone addiction on sleep quality after controlling for the effect of psychological flexibility; mediating effect value = a × b. ^***^P < 0.001.

The results of the analysis of the mediation effect showed that the upper and lower limits of the 95% confidence interval corresponding to Bootstrap for each pathway did not contain 0, indicating that psychological resilience played a statistically significant role in mediating the effect between cell phone addiction and sleep quality. Psychological resilience partially mediated the effect between cell phone addiction and sleep quality, with a mediating effect of 5.556% of the total effect size (Table 6). The mediating effect model is shown in Figure 2.

**TABLE 6:** Mediating effect test of psychological flexibility between cell phone addiction and sleep quality among college students.

	Co-eff./effect	Boot. SE	Boot 95% CI		*P*	Proportion
			Lower	Upper		
Total effect	0.054	0.002	0.050	0.059	< 0.001	
Direct effect	0.051	0.002	0.047	0.056	< 0.001	94.444%
Indirect effect	0.003	0.001	0.002	0.004	< 0.001	5.556%

## Discussion

The current study found that 16.5% of college students had sleep disorders, which is generally consistent with the data derived from previous studies (38). Descriptive analysis found that female students had higher MPAI scores and PSQI scores than male students, which is consistent with the findings of Liu and Lu (39). The study showed that females were more likely to have difficulty in falling asleep, wake up more often at night, and less sleep (40). The PSQI scores of seniors are higher than other grades, while the MPAI scores and CD-RISC scores are lower. It is possible that senior students are facing busy work in internships and employment problems after graduation, which are more psychologically stressful and prone to having sleep problems. Schools should pay more attention to senior students and give guidance in internships and employment or other aspects of life. In college students, the higher frequency of smoking and alcohol consumption, the higher the MPAI score and PSQI score, while the CD-RISC score was not good. The higher frequency of physical activity was associated with lower MPAI and PSQI scores and higher CD-RISC scores, which is consistent with the findings of Lee et al. (41, 42).

The results suggest that psychological resilience partially mediates the relationship between cell phone addiction and sleep quality among college students. The indirect effect of mobile phone addiction on sleep quality through psychological resilience accounted for 5.556% of the total effect, indicating the efficacy of the hypothetical model we constructed. Cell phone addiction predicted sleep quality directly or indirectly, and psychological resilience as a mediating variable can buffer the negative effect of cell phone addiction on sleep quality.

The study showed that college students with high MPAI scores were more likely to have poor sleep quality, which is consistent with the findings of Li et al. (43). There are several explanations for the positive association between cell phone addiction and poor sleep quality. Firstly, excessive cell phone use at bedtime may delay, replace, or disrupt the sleep process. Secondly, excessive cell phone use usually leads to higher levels of psychological stress, which also negatively affects sleep and recovery. Thirdly, the blue light emitted from the screen may affect melatonin levels, which can interfere with sleep. Finally, electromagnetic fields emitted by cell phones may also contribute to poor sleep quality (14). Among college students, cell phone addiction to some extent reflects the lack of self-control. College students are more likely to use cell phones excessively at night due to many courses and heavy tasks during the day, which shortens the sleep time. The light from cell phone screens inhibits melatonin secretion at night, disrupting the circadian rhythm of the body and leading to difficulties in falling asleep. At the same time, cell phone addiction among college students can also lead to poor concentration in class or reduced study time and study pressure, which can lead to negative emotions such as anxiety and reduced sleep quality.

To date, lots of studies have investigated risk factors for sleep quality. However, in recent years, psychological resilience (the ability to thrive in the face of adversity) has received increasing attention as a counteracting factor to stress, thus contributed greatly to recovery from adverse situations (44). Cell phones as mobile Internet carriers consume the psychological resources of college students in various ways, and their excessive use may affect emotional balance by disrupting self-control (45). Wang et al. (46) noted that cell phone addiction can lead to increased negative emotions. Similarly, some researchers have suggested that it is due to individuals’ deficits in regulating negative emotions that cell phone addiction continues to be maintained (20). Furthermore, research has shown that individuals with cell phone addiction are more likely to have difficulties with emotion regulation (47). Therefore, college students with cell phone addiction may frequently experience negative emotions and have low levels of psychological resilience. Young people with low psychological resilience are prone to having sleep disorders. On the contrary, young people with high psychological resilience are good at resolving various adverse events in life, work and study, and presenting good sleep quality (28). The reason for this may be that college students with high levels of psychological resilience can mitigate neurohormonal changes in their bodies and thus can have a positive impact on sleep quality. Another reason may be that individuals with positive psychological characteristics have a positive effect on their sleep quality by having good physical and mental health. In conclusion, psychological resilience is a protective factor for good sleep quality among college students, and can buffer the relationship between college students’ cell phone addiction and sleep quality.

This study reveals the mediating role of psychological resilience behind the relationship between college students’ cell phone addiction and sleep quality, which suggests that college educators can use emotional regulation and improved emotional control ability to solve this problem when intervening in college students’ cell phone addiction. This can be done by developing college students’ psychological resilience and tapping protective resources to buffer their pathological cell phone addiction. It can also be done by developing psychological resilience to improve the negative emotions of cell phone addicted college students and improve their sleep quality. Some studies have shown that the neurotransmitter endorphin in the brain (48) can change the negative emotions of individuals, which can reduce stress, relieve tension and bad emotional state. Certain intensity of exercise can also promote the secretion of endorphin in the brain, such as running and swimming (49). The current study also showed that the more frequent physical exercise, the lower MPAI and PSQI scores and the higher CD-RISC scores, which fully proved the benefits brought by proper exercise. College psychologists can also guide college students to use cognitive reappraisal (50) (change the understanding of emotional events and change the perception of their personal meaning) through individual counseling or group counseling to reduce their reactions to and experiences of negative events (cell phone addiction). They can guide them to maintain their psychological resilience through reasonable regulation of emotional catharsis, reduce college students’ dependence on mobile phones and improve their sleep status. Last but not least, this study was implemented during the COVID-19 pandemic, and the epidemic had an impact on the physical and mental health as well as the sleep status of college students over the past 3 years. Acute stress, anxiety and depressive symptoms have been prevalent among college students during the COVID-19 epidemic (51, 52). Therefore, future investigations are necessary to validate the conclusions drawn from this study.

## Limitations

There are several limitations to our study. Firstly, this was a sample based on a population of college students, which may be the main limitation comparing to other studies. Further research could expand the scope of the study population. Secondly, the data are just from one province, the results and conclusions may be influenced by local sociological factors. There is a serious cause for constant stress due to the very strict COVID-19 regulations in China, the comparison to other regions will be limited, and our results might be hardly transferable to other countries. Finally, our current study only considered psychological resilience, but there are still other factors that may mediate the association between cell phone addiction and sleep quality. Therefore, we suggest that future studies can consider anxiety, loneliness, and depression. Despite these limitations, this study examined the parallel mediating role of psychological resilience on the relationship between cell phone addiction and sleep quality. It also provides new intervention strategies from a psychological resilience perspective to buffer the negative effects of cell phone addiction on sleep quality.

## Data availability statement

The original contributions presented in this study are included in this article/supplementary material, further inquiries can be directed to the corresponding author.

## Ethics statement

The studies involving human participants were reviewed and approved by the Ethics Committee of Xuzhou Medical University. The patients/participants provided their written informed consent to participate in this study.

## Author contributions

QW and XG analyzed the data. QW, GX, JZ, and DY conceived and designed the study. QW drafted the manuscript. DY reviewed and made improvements in the manuscript. All authors read and approved the final manuscript.

## Footnotes

1
www.wjx.cn
